# Suppressed *de novo* lipogenesis by plasma membrane citrate transporter inhibitor promotes apoptosis in HepG2 cells

**DOI:** 10.1002/2211-5463.12435

**Published:** 2018-05-14

**Authors:** Phornpun Phokrai, Wan‐angkan Poolsri, Somrudee Suwankulanan, Narinthorn Phakdeeto, Worasak Kaewkong, Dumrongsak Pekthong, Lysiane Richert, Piyarat Srisawang

**Affiliations:** ^1^ Department of Medical Technology Faculty of Science and Technology Bansomdejchaopraya Rajabhat University Bangkok Thailand; ^2^ Department of Physiology Faculty of Medical Science Naresuan University Phitsanulok Thailand; ^3^ Department of Biochemistry Faculty of Medical Science Naresuan University Phitsanulok Thailand; ^4^ Department of Pharmacy Practice Faculty of Pharmaceutical Sciences Naresuan University Phitsanulok Thailand; ^5^ Kaly‐Cell Plobsheim France

**Keywords:** apoptosis, *de novo* lipogenesis, HepG2 cells, plasma membrane citrate transporter

## Abstract

Suppression of the expression or activities of enzymes that are involved in the synthesis of *de novo* lipogenesis (DNL) in cancer cells triggers cell death via apoptosis. The plasma membrane citrate transporter (PMCT) is the initial step that translocates citrate from blood circulation into the cytoplasm for *de novo* long‐chain fatty acids synthesis. This study investigated the antitumor effect of the PMCT inhibitor (PMCTi) in inducing apoptosis by inhibiting the DNL pathway in HepG2 cells. The present findings showed that PMCTi reduced cell viability and enhanced apoptosis through decreased intracellular citrate levels, which consequently caused inhibition of fatty acid and triacylglycerol productions. Thus, as a result of the reduction in fatty acid synthesis, the activity of carnitine palmitoyl transferase‐1 (CPT‐1) was suppressed. Decreased CPT‐1 activity also facilitated the disruption of mitochondrial membrane potential (ΔΨm) leading to stimulation of apoptosis. Surprisingly, primary human hepatocytes were not affected by PMCTi. Increased caspase‐8 activity as a consequence of reduction in fatty acid synthesis was also found to cause disruption of ΔΨm. In addition, apoptosis induction by PMCTi was associated with an enhanced reactive oxygen species generation. Taken together, we suggest that inhibition of the DNL pathway following reduction in citrate levels is an important regulator of apoptosis in HepG2 cells via suppression of CPT‐1 activity. Thus, targeting the DNL pathway mediating CPT‐1 activity by PMCTi may be a selective potential anticancer therapy.

**Abbreviations**
ACC: acetyl‐CoA carboxylase
ACLY: ATP‐citrate lyase
CPT‐1: carnitine palmitoyl transferase‐1
DNL: *de novo* lipogenesis
FASN: fatty acid synthase
HCC: hepatocellular carcinoma
LCFAs: long‐chain fatty acids
OXPHOS: oxidative phosphorylation
PMCTi: PMCT inhibitor
PMCT: plasma membrane citrate transporter
ROS: reactive oxygen species
TOFA: 5‐(tetradecyloxy)‐2‐furoic acid

In cancer cells, the Warburg effect has been recognized as a reprogrammed metabolism pathway which features an increase in both glucose uptake and switching of pyruvate to lactate even in the presence of enough oxygen, leading to a state termed ‘aerobic glycolysis’ 1, 2. Regardless of the presence of an oxygen supply, ATP generation via the mitochondrial oxidative phosphorylation (OXPHOS) pathway has been reported to concomitantly reduce in many cancer cells 3, 4, 5. Meanwhile, cancer growth and prognosis are associated with an upregulation of the *de novo* lipogenesis (DNL) pathway. The newly biosynthesized long‐chain fatty acids (LCFAs) in the DNL pathway serve ATP through mitochondrial β‐oxidation and molecule building blocks required for biosynthesis of constituents of cell membrane phospholipids and cellular signaling proteins 6, 7, 8. The initial step of the DNL pathway is enzymatic conversion of cytoplasmic citrate into acetyl‐CoA for the sequential synthesis of LCFAs. In contrast to cancer cells, the DNL pathway is usually minimal in normal cells of which their synthesis is dependent on nutritional supply 9.

The synthesis of *de novo* fatty acids starts with citrate in the cytosol which is converted to acetyl‐CoA by ATP‐citrate lyase (ACLY). Acetyl‐CoA carboxylase (ACC) then catalyzes acetyl‐CoA to malonyl‐CoA, and fatty acids synthase (FASN), the main biosynthetic enzyme, performs the conversion of malonyl‐CoA to LCFAs, especially 16‐C palmitate. The new targeting of cancer therapies has focused on DNL enzymes to become one of the potential new chemotherapy drugs 10. Previous research has reported that the inhibition of ACC reduces tumor growth of pancreatic cancer xenografted in animal models 11 and increases apoptosis in glioblastoma cell lines 12. Following small interfering RNA‐mediated inhibition of ACLY in MCF‐7 breast cancer cells, cell viability was suppressed and cell apoptosis was increased 13. Additional supporting evidence to verify the DNL pathway in regulating cancer cell viability has been shown where the inhibition of FASN by FASN inhibitor‐alcohol extract of clove suppressed the S‐phase DNA replication of HepG2 cells 14. The level of fatty acid is known to exert positive inhibitory feedback on ACC activity 15. The malonyl‐CoA product from ACC activity regulates mitochondrial carnitine palmitoyl transferase‐1 (CPT‐1) activity which involves conversion of fatty acid into fatty acyl CoA for β‐oxidation. Alteration of β‐oxidation has been shown to correlate with apoptosis in cancer cells 16, 17, 18. Thus, inhibition of the DNL pathway results in an antiproliferative effect in cancer cells, suggesting an effective treatment of cancer cells by targeting the DNL pathway. In addition, targeted molecular therapies have nowadays emerged and developed as alternative options to overcome the limited efficacy of common therapeutic approaches.

The cytoplasm citrate pool serves as an important precursor for fatty acid synthesis in the DNL pathway 19, 20. It is known that there exist at least two sources of citrate in cytoplasm. The first one is the influx of plasma citrate by the plasma membrane citrate transporter protein (PMCT) or SLC13A5 or Na^+^‐dependent citrate transporter (NaCT). The other is the efflux of output from the mitochondrial tricarboxylic acid (TCA) cycle by the mitochondrial citrate transport protein (CTP) or SLC25A1 or citrate carrier (CiC) 20. Previous studies have suggested that citrate flux from the TCA cycle in mitochondria to cytoplasm via CTP is increased in Morris hepatoma 3924A and hepatoma 16, liver cancer cells, compared with normal cells 21. CTP has been shown to regulate cell survival and is also essential for mitochondrial homeostasis in human lung and breast cancer 22, 23. Meanwhile, the expression of PMCT is predominately in the plasma membrane of a mammalian normal liver and at lower levels in the brain and testes 24. Data of hepatic citrate influx studied in humans and rodents indicate that PMCT plays an important role in citrate clearance from plasma 25.

PMCT was originally discovered in *Drosophila melanogaster* (*D. melanogaster*), known as INDY (I'm not dead yet) and *Caenorhabditis elegans* (*C. elegans*), known as NAC‐2. A reduction in INDY activity in *D. melanogaster* and functional knockdown of NAC‐2 by RNAi in *C. elegans* have a similar effect, leading not only to a significant increase in average life span, but also causing a significant decrease in body size and fat content without an alteration of either physical activity or fertility 20, 26. Recent study has also confirmed that citrate transported by mammalian INDY (mINDY) contributes in regulation of liver fat metabolism 27. Inhibition of mINDY reduced hepatic lipid accumulation, improved hepatic insulin sensitivity, and prevented diet‐induced nonalcoholic fatty liver disease in adult C57BL6/J mice 27. Thus, PMCT has been proposed as a therapeutic target for obesity and diabetes 28. In addition, the expression levels of genes using RNA‐Seq (RNA sequencing) data obtained from the web database of the Gene Expression Atlas at https://www.ebi.ac.uk/gxa/ reported that human RNA levels of SCL13A5 or PMCT are highly expressed in hepatocellular carcinoma (HCC) HepG2 cells which is often used to study the fatty liver‐induced HCC model 29, 30. However, the role of PMCT in fatty acid synthesis in cancer cells was not further evaluated. Thus, taken together, all this evidence suggests that both CTP and PMCT orchestrate the regulation of fatty acid synthesis in cancer cells. We hypothesized that targeting the DNL pathway in cancer cells through inhibition of citrate transporters may offer a potential anticancer therapy. This study aimed to evaluate the mechanism of the PMCT inhibitor (PMCTi), which might be involved in the deficiency of the fatty acid levels via the DNL pathway in HepG2 cells. This effect led to induction of cancer cell apoptosis.

## Materials and methods

### Chemicals and reagents

The PMCTi was purchased from TimTec (Harmony Business Park, Newark, DE, USA). Eagle's minimum essential medium (EMEM), Dulbecco's modified Eagle's medium (DMEM; Corning, Manassas, VA, USA), and all other chemicals used in this study were obtained from Corning, Life Technologies (Grand Island, NY, USA), and Sigma Chemical Co. (St. Louise, MO, USA).

### Cell culture

Human HCC, HepG2 cells, was purchased from American Type Culture Collection (ATCC number: HB‐8065) (Manassas, VA USA), and HuH‐7 cells were purchased from Japanese Collection of Research Bioresources Cell Bank (JCRB0403; JCRB, Osaka, Japan). KKU‐055 cells, human poorly differentiated cholangiocarcinoma established from the biliary tract, were provided by Cholangiocarcinoma Research Institute, Faculty of Medicine, Khon Kaen University, Thailand. HepG2 cells were cultured in EMEM, while HuH‐7 and KKU‐055 cells were cultured in DMEM media. The complete growth medium was prepared containing 10% fetal bovine serum, 3 mm l‐glutamine, 100 μg·mL^−1^ streptomycin, and 100 μg·mL^−1^ penicillin. Cells were incubated at 37 °C in 5% CO_2_. The cultures were passaged at least two times a week.

### Cell viability assay by MTT

To assess cell viability, cells were plated in 96‐well plates and incubated overnight. Then, cells were incubated with fresh EMEM medium containing different concentrations of PMCTi for 24 h and vehicle controls were included for each plate. After 24 h of incubation at 37 °C, the medium was removed before MTT solution was added into each well, and cells were incubated at 37 °C. The reagent was removed carefully, and MTT‐formazan crystals were dissolved in DMSO at room temperature. Absorbance was measured at 595 nm with the multi‐well microplate reader (program gen 5 version 2.00.18 Biotek Reader Photometer, Biotek Instruments, Inc., Winooski, VT, USA).

### Apoptosis assay by flow cytometry

After PMCTi treatment overnight, cells were harvested and resuspended in 1X annexin‐binding buffer. Then, the pellets were stained with Alexa Fluor 488 Annexin V and PI working solution. Cells were incubated at room temperature in the dark for 15 min, and then, the samples were kept on ice. The stained cells were analyzed by flow cytometry to measure fluorescence emission by FACScalibur flow cytometry (Becton Dickinson, Franklin Lakes, NJ, USA), and the data were analyzed using cellquestpro software (BD).

### Intracellular citrate quantification assay

Intracellular citrate levels were detected by the Citrate Assay Kit (Abcam, Biomed Diagnostics (TH) Co., Ltd, Bangkok, Thailand). Briefly, HepG2 cells were seeded in a 60‐mm^3^ petri dish and allowed an overnight period of attachment. Citrate was formed by the addition of oxaloacetate to the acetyl group of acetyl‐CoA derived from the glycolysis pathway. Citrate was converted to pyruvate which was then quantified by fluorescence intensity measured at Ex/Em 535/590 nm using the Synergy HT Microplate Reader with gen5 data analysis software (Biotek Instruments, Inc.). Results were expressed as a percentage of intracellular citrate compared with the control.

### Intracellular free fatty acid quantification assay

FASN activity was determined on cellular extracts of HepG2 cells by detection of the product of the *de novo* fatty acid synthesis, using the Free Fatty Acid Quantification Kit (Abcam). After 24 h of treatment, HepG2 cells were harvested and homogenized with chloroform/Triton X‐100 solution. After removing chloroform from the sample, acetyl‐CoA synthase reagent was added to the sample to convert LCFAs to CoA derivatives. Then, the reaction mix containing assay buffer, enzyme mix, enhancer, and fluorescence probe was added to measure the fluorescence at Ex/Em 535/590 nm using the Synergy HT Microplate Reader. Results were expressed in percentage of intracellular LCFA compared with the control.

### Intracellular triacylglycerol quantification assay

The triacylglycerol concentration in HepG2 cells after treatment with PMCTi was determined using triacylglycerol quantification bioassay kit (T8417; US Biological; Life Sciences, Salem, MA, USA). In the assay, triacylglycerol were converted to free fatty acids, and glycerols were oxidized to generate product which was measured by fluorometric method. Briefly, 24 h after treatment, cells were harvested and homogenized with Triton X‐100. The sample was slowly heated from 50 °C to 80 °C in a dry block heater two times. The supernatant was collected by centrifugation to remove insoluble materials. The lipase and the reaction mix, including probe and assay buffer were added to each sample and incubated for 60 min at room temperature in the dark, followed by detection of the fluorescence at Ex/Em 535/590 nm using the Synergy HT Microplate Reader. Results were expressed in percentage of intracellular triacylglycerol compared with the control.

### Immunoblotting analysis

After treatment with PMCTi for 24 h, HepG2 cells were trypsinized and lysed with lysis buffer (M‐PER mammalian protein extraction reagent), containing protease inhibitors (Thermo Scientific, Pierce, IL, USA). Protein content of the cell lysate was determined by BCA protein assay reagent (Thermo Scientific). Extracted proteins were subjected to 8‐12% SDS‐polyacrylamide gel. Following electrophoresis, protein blots were then transferred to poly(vinylidene difluoride) membrane (Merck Millipore, Darmstadt, Germany). After blocking of nonspecific binding, the membrane was subsequently incubated overnight at 4 °C with primary antibodies against FASN (Biomed Diagnostics (TH) Co., Ltd), ACC (Millipore, Temecula, CA, USA), and ACLY (Cell Signaling Technology, Danvers, MA, USA) (Dilution 1 : 1000). The membranes were incubated with horseradish peroxidase‐conjugated goat anti‐Rabbit IgG secondary antibody (Life Technologies) diluted 1 : 5000 in 0.2% Tween‐20 PBS solution containing 5% nonfat dry milk for 2 h at 4 °C. The blots were developed using Clarity Western ECL Chemiluminescent Substrate Reagent Kit (Bio‐Rad Laboratories Ltd., Pathumwan, Bangkok, Thailand). β‐Actin was also measured as a loading control. The intensity of each band was determined using the CCD camera (ImageQuant LAS 4000; GE Healthcare Life Sciences, Pittsburgh, PA, USA). The fluorescence imaging of cellular protein expression was also detected by a confocal laser scanning microscope (OLYMPUS FV1000, Center of Nano Imaging, CNI, and Olympus Bioimaging Center, OBC, Mahidol University, Thailand) using DAPI as a counter stain.

### Mitochondrial membrane potential (∆Ψm) detection by flow cytometry

The level of ∆Ψm in HepG2 cells was measured by flow cytometry using 5,5′,6,6′‐tetrachloro‐1,1′,3,3′‐tetraethylbenzimidazolcarbocyanine iodide (JC‐1) Dye‐Mitochondrial Membrane Potential Probe (Life Technologies) in accordance with the manufacturer's suggested protocol. Briefly, cells were plated in a cell culture dish 60 mm^3^. After 24 h of treatment, cells were washed in PBS, harvested, and resuspended with warm PBS buffer. JC‐1 dye was then added to each sample tubes and incubated at 37 °C with 5% CO_2_ for 45 min. Carbonyl cyanide m‐chlorophenylhydrazone (CCCP) was used as a positive control of the loss of ∆Ψm and to verify the response of JC‐1 to alteration of ∆Ψm. After being labeled with JC‐1 dye, cells were analyzed by FACScalibur flow cytometry. A monomeric JC‐1 form existing in cytoplasm emits green fluorescence intensity (~529 nm). An aggregated JC‐1 form accumulated in mitochondria emitted red fluorescence intensity (~590 nm). Data were analyzed using cellquestpro software (BD). The fluorescence imaging of ∆Ψm was detected by a confocal laser scanning microscope using DAPI as a counter stain.

### Determination of carnitine palmitoyl transferase‐1 (CPT‐1) activity

CPT‐1 activity was quantified by spectrophotometric method as described previously by Kant *et al*. 31. Briefly, following PMCTi‐treated cells, mitochondrial proteins were isolated by lysis buffer (Tris/HCl pH 7.4) containing 0.25 mm sucrose and 1 mm EDTA. The samples were centrifuged at 500 ***g*** at 4 °C for 10 min, re‐centrifuged at 15 000 ***g*** at 4 °C for 15 min, and mitochondria proteins were resuspended in lysis buffer. The mitochondria protein content was determined. The reaction mix containing Tris‐buffer (100 mm, pH 8.0, 0.1% Triton X‐100, 1 mm EDTA), 0.01 mm palmitoyl CoA, and 0.5 mm DTNB was added in each sample followed by measurement of the absorbance at 412 nm using a microplate reader. Then, l‐carnitine at 1.25 mm was added, incubated for 5 min, and measured for absorbance at 412 nm.

### Determination of caspase‐8 activity

Caspase‐8/FLICE Colorimetric Assay Kit (PK‐CA577‐K112‐100; PromoKine, Heidelberg, Germany) was used to determine the activity of caspase‐8 that recognizes the sequence IETD (IIe‐Glu‐Thr‐Asp) and based on spectrophotometric detection of the chromophore p‐nitroanilide (pNA) after cleavage from the labeled substrate IETD‐pNA and then quantified using a fluorescence microplate reader at Ex/Em 360/460 nm.

### The reactive oxygen species (ROS) generation assay

Reactive oxygen species generation, including hydrogen peroxide, hydroxyl radicals, and peroxynitrite, was analyzed by flow cytometry. HepG2 cells were treated with PMCTi for 24 h and then stained with fluorescent dye 5‐(and‐6)‐chloromethyl‐2′,7′‐dichlorodihydrofluorescein diacetate, acetyl ester (CM‐H_2_DCFDA). This dye probe diffuses through a cell membrane and is hydrolyzed by intracellular esterase to dichlorodihydrofluorescein, which then is oxidized to dichlorofluorescein, a fluorescent product. The percentage of ROS level was investigated by flow cytometry. Data were analyzed using cellquestpro software (BD). ROS generation was imaged by confocal laser scanning microscopy using DAPI as a counter stain.

### Statistical analysis

The results were analyzed by *t*‐test and one‐way ANOVA using a Turkey test as a post‐test. *P* < 0.05 versus the control was considered statistically significant using graph prism Software, version 5 (GraphPad Software, Inc., La Jolla, CA, USA). At least three independent experiments were performed for statistical analysis and expressed as mean ± SEM.

## Results

### The effects of PMCTi on inhibition of cancer cell viability

**Figure 1:**
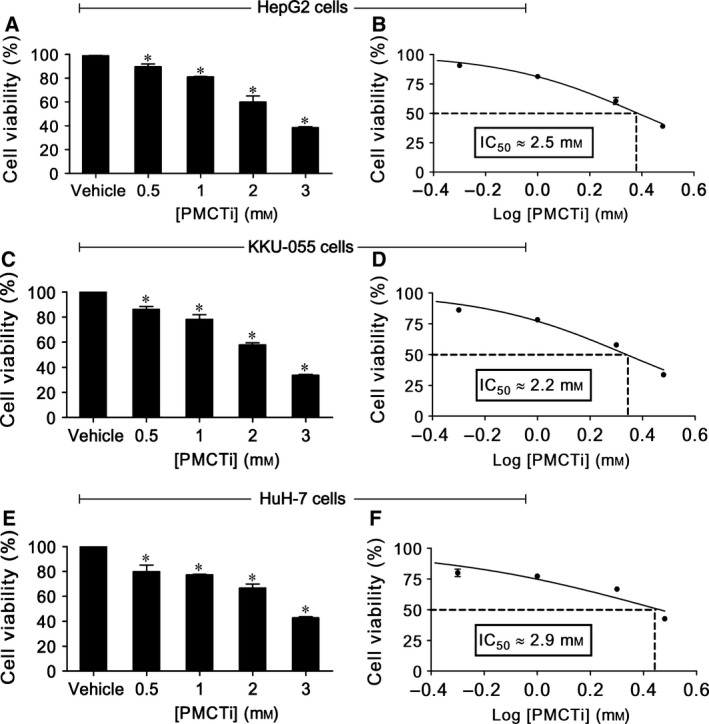
Effect of PMCTi on cell viability of cancer cells. Cells were incubated with different concentrations (0.5–3 mm) of PMCTi for 24 h. MTT assay was performed to determine the number of viable cells compared with 100% of the control (A, C, E), and IC_50_ values were expressed (B, D, F). The control was defined as cells treated with a medium or 0.2% DMSO vehicle without an inhibitor. Data are presented as mean ± SEM from at least three independent experiments performed in triplicate, *P < 0.05 significantly different from the control.

The MTT assay is a colorimetric cytotoxicity assay and one of the most widely employed viability assays in biomedical research 32. This assay is a cost effective and rapid method for investigating cell viability or proliferation 33. The reduction reaction of the tetrazolium compound to formazan by cellular enzymes, including mitochondrial NAD(P)H‐dependent oxidoreductases and dehydrogenases, is lowered in a population of cells exposed to a toxic compound 34. To investigate that PMCTi suppressed cell viability, HepG2, KKU‐005, and HuH‐7 cells were used. MTT assay was performed after cells were treated with 0.5–3 mm of PMCTi for 24 h. Figure 1A,C,E show that PMCTi induced a significant reduction in the number of viable cells when compared with the control in a dose‐dependent manner. Figure 1B,D,F shows IC_50_ of PMCTi treatment; HepG2, KKU‐005, and HuH‐7 cells were approximately 2.5, 2.2, and 2.9 mm, respectively.

Previous study has reported that the liver cancer cells exhibit the highest PMCT expression, especially in HepG2 cells, which is consistent with data obtained from the web database of Human Protein Atlas (http://www.proteinatlas.org). Therefore, HepG2 cells having an IC_50_ lower than HuH‐7 cells and a little higher than KKU‐005 cells were used to study the underlying mechanisms of apoptosis cell death from the effects of PMCTi.

### The effects of PMCTi on apoptosis induction in HepG2 cells

**Figure 2:**
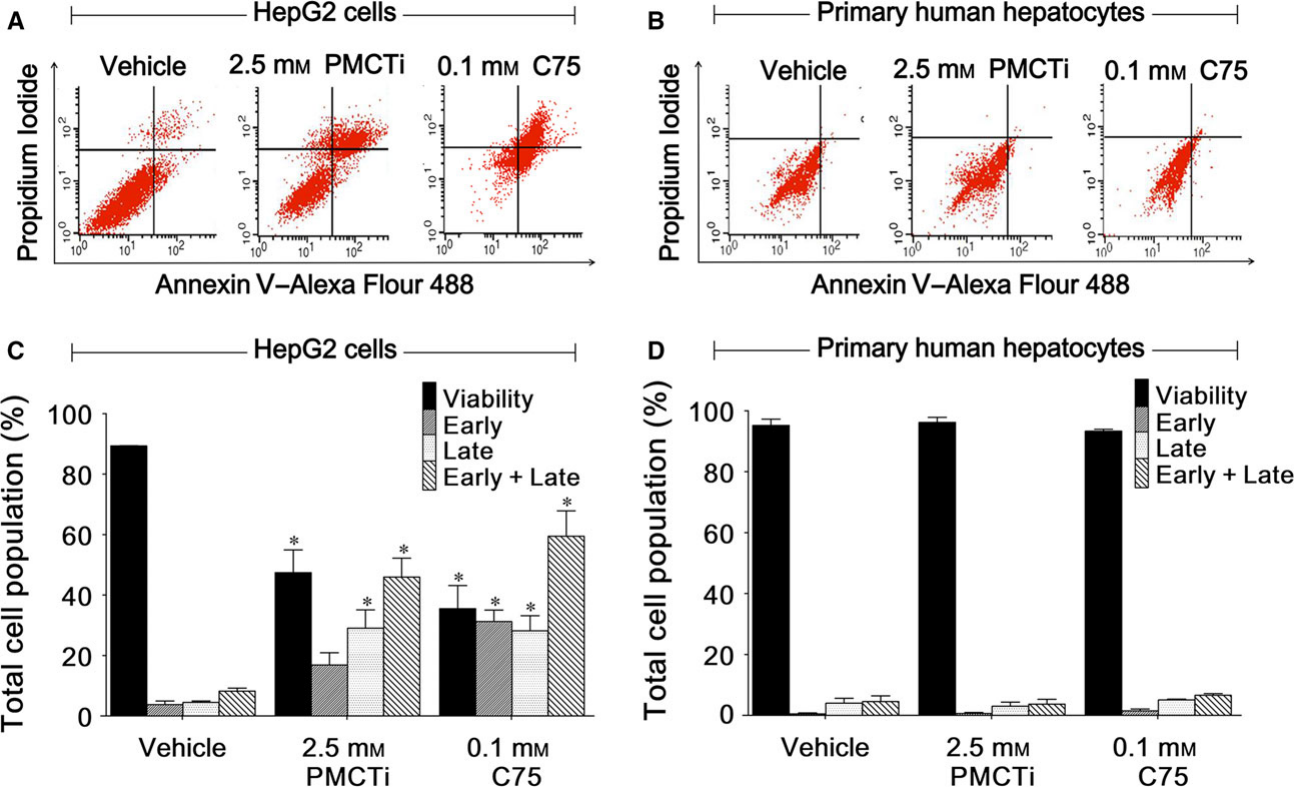
Effect of PMCTi on apoptosis induction of HepG2 cells. (A,B) Representative flow cytometry scatterplots are depicted using double staining with Annexin V–Alexa Fluor 488 and propidium iodide (PI) to determine the distribution of viable, early, and late apoptotic cells in HepG2 cells and primary hepatocytes, respectively. (C,D) The percentages of cells relative to the whole cell populations (set as 100%) are expressed by bar charts showing the proportion of viable, early, late, and early plus late apoptotic cells in HepG2 cells and primary hepatocytes, respectively. C75 was used as a known apoptotic induction compound. The control was defined as cells treated with a medium or 0.2% DMSO vehicle without an inhibitor. Data are presented as mean ± SEM from at least three independent experiments performed in triplicate, *P < 0.05 significantly different from the control.

To examine whether the mechanism of PMCTi inducing cell death was due to apoptosis in HepG2 cells, cells were treated with 2.5 mm PMCTi for 24 h, stained with Alexa Fluor 488 Annexin V and PI, and detected by flow cytometry. As shown in Fig. 2A,C, the control group, cells exposed to medium or vehicles alone, had negative staining of both Annexin V and PI. This staining indicated the number of viable cells and cells in apoptotic stages. The early apoptotic cell was assessed by the cell having a positive staining with Annexin V but negative staining with PI, while the late apoptotic cell was observed to be stained with both Annexin V and PI. After 24 h of incubation with 2.5 mm of PMCTi, the rate of apoptosis in HepG2 cells was increased when compared with the control group. This result demonstrates that PMCTi induces apoptosis in HepG2 cells. Figure 2B,D shows that PMCTi did not cause apoptosis in primary human hepatocytes. Consistently, apoptotic induction of 0.1 mm C75 for 24 h was markedly increased in HepG2 cells but not in primary human hepatocytes.

### The effect of PMCTi on DNL protein expressions, citrate, fatty acid, and triacylglycerol levels in HepG2 cells

**Figure 3:**
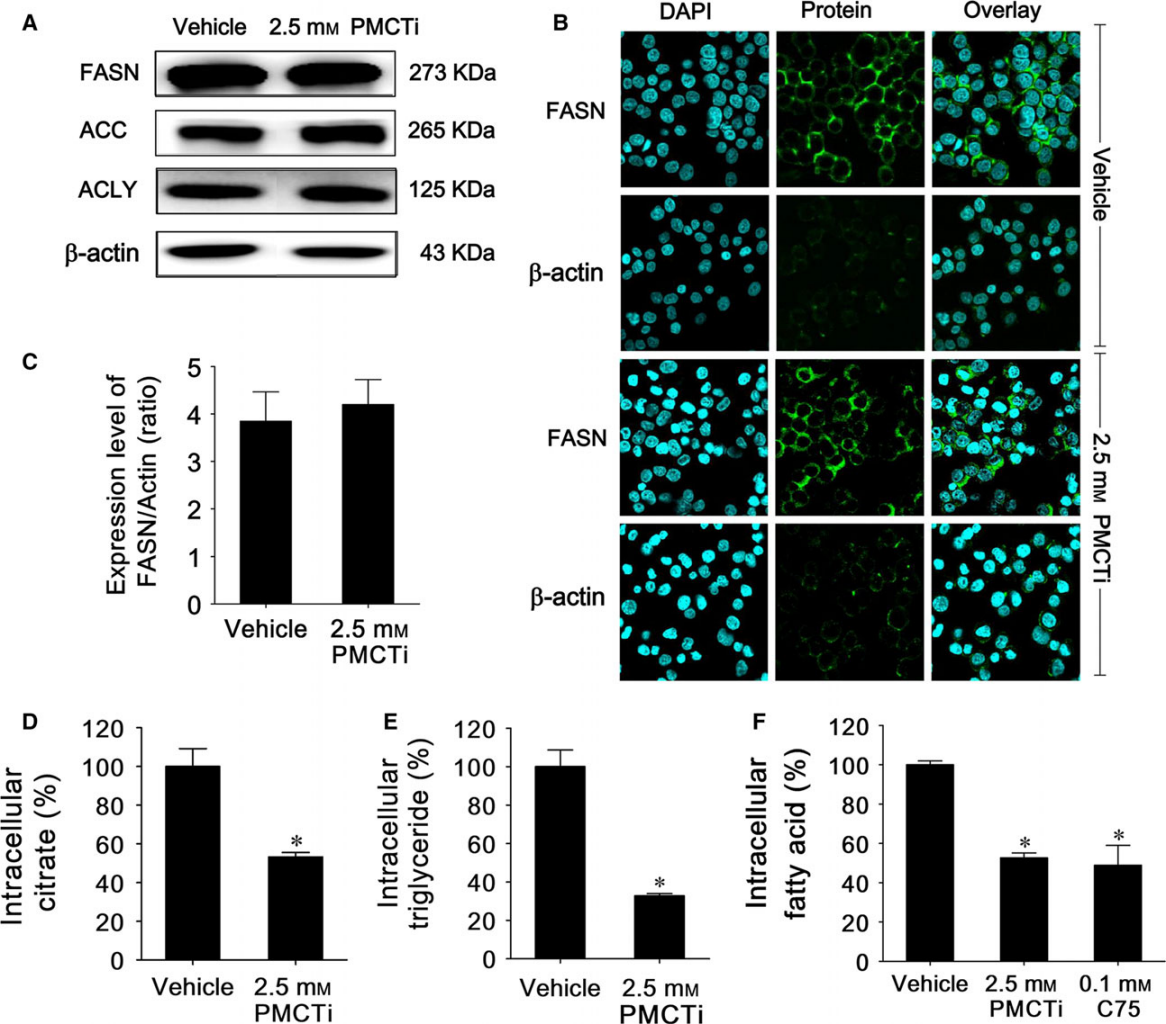
The effects of PMCTi treatment on the *de novo* lipogenesis pathway in HepG2 cells. (A) Lipogenic protein ACLY, ACC, and FASN expressions were detected by immunoblot, and (B) representative FASN protein expression was observed under confocal laser scanning microscopy (OLYMPUS FV1000). The control was defined as cells treated with a medium or 0.2% DMSO vehicle without an inhibitor. The band of proteins was representative of those obtained from at least three independent experiments. β‐Actin was used as an internal control for integrity with an equal amount of protein loading and transferring. (C) Bar graphs show the relative expression ratio of FASN/β‐actin. (D) Citrate, (E) fatty acid, and (F) triacylglycerol levels were detected and quantified in percentage compared with 100% of the vehicle control. Data are presented as mean ± SEM from at least three independent measurements performed in triplicate, *P < 0.05 significantly different from the control.

It has been reported that suppression of the DNL pathway is a potential target for inhibiting cancer cells 12, 35, and thus, this study speculated that PMCTi might suppress the DNL pathway, which could lead to apoptosis induction in HepG2 cells. We investigated the inhibitory effect of 2.5 mm of the PMCTi for 24 h on the expression of ACLY, ACC, and FASN. As shown in Fig. 3A–C, there was no alteration in the abundance of all three protein expressions compared with the control by PMCTi treatment. Remarkably, decrease in intracellular citrate (Fig. 3D) following PMCTi treatment was concomitantly observed with a decrease in both intracellular fatty acid and triacylglycerol levels (Fig. 3E,F). The percentage of intracellular citrate after cells were treated with 2.5 mm of PMCTi for 24 h was decreased to 51.23%, and the percentage of intracellular free fatty acid and triacylglycerol was decreased to 56.23% and 32.72%, respectively, compared with 100% of the control. This study demonstrates that inhibition of the DNL pathway might be a potential cause of apoptosis in HepG2 cells. PMCTi depletes intracellular substrate for the DNL pathway, which leads to decreased fatty acid and triacylglycerol products without changes in the abundance of DNL protein expressions.

### The effects of PMCTi on disruption of mitochondrial membrane potential (∆Ψm) in HepG2 cells

**Figure 4:**
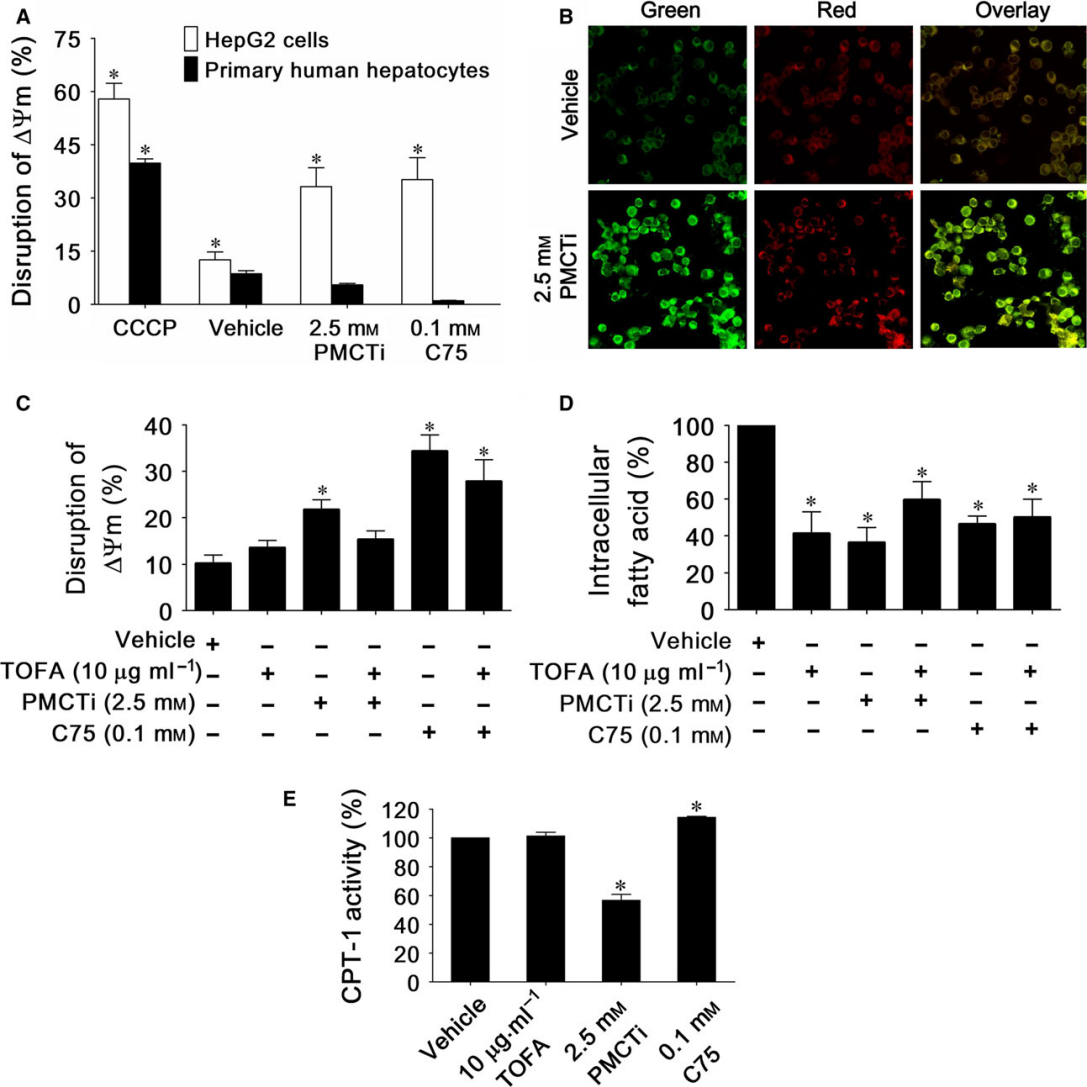
The effect of PMCTi inducing malonyl‐CoA accumulation leading to CPT‐1 inhibition is responsible for disruption of mitochondrial membrane potential (∆Ψm) in HepG2 cells. The ∆Ψm was evaluated by calculating the relative level of red (J‐aggregated form) to green (J‐monomeric form) fluorescence intensity from a flow cytometry analysis. (A) The percentage of disruption of ∆Ψm in HepG2 cells relative to the whole cell population is expressed in bar charts. Cells were incubated with CCCP as a positive control of the depolarization of the mitochondrial membrane. (B) The ∆Ψm of HepG2 cells treated with vehicle or PMCTi were stained with JC‐1 fluorochrome and examined by inverted confocal laser scanning microscopy. (C) From the flow cytometry experiment, pretreatment 1 h with 10 mg·mL^−1^ TOFA reduced disruption of ∆Ψm observed following the treatment with 12 h PMCTi. (D) The percentage of intracellular fatty acid expressed in bar charts shows a reduction in fatty acid level in cells which were pretreated with TOFA followed by the treatment with 12‐h PMCTi. (E) Bar charts represent the CPT‐1 activity expressed as percentage compared with 100% of vehicle control. Data are presented as mean ± SEM from at least three independent experiments performed in triplicate, *P < 0.05 versus control.

Depolarization of the mitochondrial membrane potential is a special characteristic of the initiation event of the intrinsic and extrinsic mitochondrial‐dependent apoptotic pathway 36. Depletion of the DNL pathway causes apoptosis via the mitochondrial‐dependent pathway in cancer cells 37. Thus, this study further examined PMCTi inducing apoptosis in HepG2 cells, which involved the disruption of ∆Ψm. The JC‐1 fluorescence dye was used as an indicator of ∆Ψm status. In healthy cells, the mitochondria have a high membrane potential with JC‐1 forming complexes in mitochondria. Accumulation in the mitochondria of dye emits a red fluorescence and is detected by flow cytometry. With the loss of ∆Ψm or depolarization in apoptotic cells, JC‐1 remains in the monomeric form and accumulates in the cytoplasm, which emits green fluorescence. The relative ∆Ψm value was obtained by calculating the ratio between levels of red to green fluorescence. A relative decrease in the red/green fluorescence intensity ratio was therefore considered depolarization of ∆Ψm. Figure 4A shows that cells with depolarization of ∆Ψm induced by 2.5 mm of PMCTi for 24 h presented an increase in green fluorescence intensity to 33.18% when compared with approximately 10% of the vehicle control. Cells treated with CCCP at 50 μm were used as a positive control to induce an increase in green fluorescence intensity, indicating a decrease in ∆Ψm. The level of ∆Ψm showed no significant alteration following PMCTi treatment for 24 h in primary human hepatocytes. C75 at 0.1 mm treatment for 24 h in HepG2 cells had a significant increase in the disruption of ∆Ψm but not in primary human hepatocytes. Figure 4B shows an increased ∆Ψm in HepG2 cells treated with 2.5 mm PMCTi for 24 h, which were imaged by confocal fluorescence microscopy.

### The effect of PMCTi on reduction in CPT‐1 activity, which is a consequence of an accumulation of malonyl‐CoA following reduction in fatty acid level in HepG2 cells

*De novo* fatty acids play an important role as a negative inhibitor on ACC activity that consequently controls carnitine palmitoyl transferase I (CPT‐1) activity 38. Increase in malonyl‐CoA level as a result of ACC inhibition consequently competitively suppresses CPT‐1 activity on catalyzing the transport of LCFAs for mitochondrial β‐oxidation. This leads to a contribution to apoptosis in cancer cells 39. We therefore evaluated the mechanism of PMCTi‐induced apoptosis in HepG2 cells. Suppression of fatty acid synthesis by PMCTi was hypothesized to enhance malonyl‐CoA accumulation which resulted in inhibition of CPT‐1 activity. Apoptosis was a consequence of this inhibition. Apoptosis was quantified by JC‐1 dye staining, which detected the status of ∆Ψm, using flow cytometry. The percentage of the control group stained with JC‐1 showed only 10% green intensity, indicating cells were in high ΔΨm. Cells with depolarization of ΔΨm were found following induction of 2.5 mm of PMCTi for 12 h. Green fluorescence intensity increased to 21.64%. Pretreatment with TOFA (5‐tetradecyloxy‐2‐furoic acid), an ACC inhibitor, followed by PMCTi treatment significantly restored ∆Ψm to normal, as shown in Fig. 4C. In addition, reduction in fatty acid was accompanied with PMCTi pretreatment with TOFA (Fig. 4D). TOFA alone that had a low fatty acid level was nontoxic to ΔΨm in HepG2 cells. We suggest that reduced fatty acid accompanied by accumulated malonyl‐CoA plays a role in apoptosis induction in PMCTi treatment. Inhibition of either malonyl‐CoA or fatty acid synthesis alone by TOFA was not a key factor in regulating apoptosis in HepG2 cells.

We further evaluated inhibition of CPT‐1 activity as a result of malonyl‐CoA accumulation in 24‐h PMCTi treatment. Inhibition of CPT‐1 activity triggers apoptosis in many cancer cells 39, 40, 41. Figure 4E shows that inhibition of both malonyl‐CoA and fatty acid synthesis by TOFA had no inhibitory effect on CPT‐1 activity. This result confirms that PMCTi treatment caused approximately 43% inhibition on CPT‐1 activity in HepG2 cells. This resulted from the inhibitory effect of reduced fatty acid accompanied by accumulated malonyl‐CoA following PMCTi treatment. Moreover, inhibition of malonyl‐CoA synthesis by TOFA had no amelioration effect of C75 on disruption of ΔΨm in HepG2 cells. Depletion of fatty acid synthesis by C75 contributes to apoptosis independently on malonyl‐CoA suppressed CPT‐1 activity 42. Thus, C75 causes a direct stimulating effect on CPT‐1 activity in HepG2 cells 41.

### The effect of PMCTi on reactive oxygen species (ROS) generation and enhanced caspase‐8 activity in HepG2 cells

**Figure 5:**
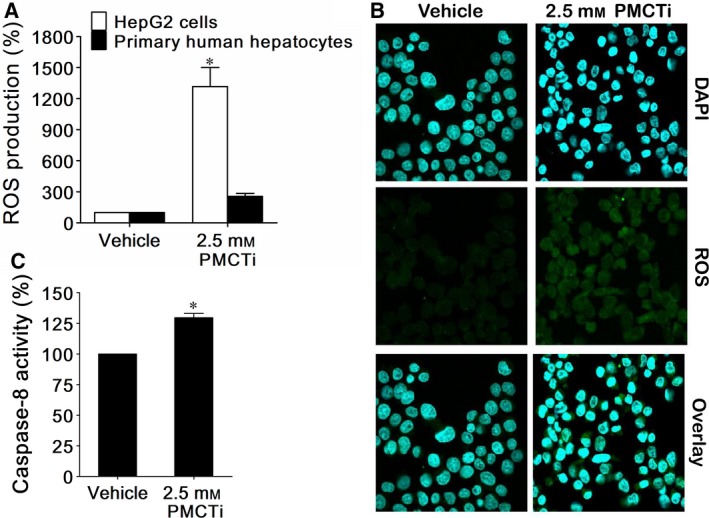
PMCTi increased ROS levels in HepG2 cells. After incubation of 2.5 mm of PMCTi for 24 h, (A) HepG2 cells and primary human hepatocytes were stained with CM‐H_2_ DCFDA, determined by flow cytometry, and expressed in percentage of ROS production compared with 100% of the control. (B) Representative imaging of ROS generation was detected under a confocal microscope. The blue channel showed DAPI‐stained nuclei, and the green channel showed CM‐H_2_ DCFDA‐expressed dichlorofluorescein. (C) Caspase‐8 activity was measured and expressed as percentage of the control. The control was defined as cells treated with a vehicle without an inhibitor. Data are presented as mean ± SEM, *P < 0.05 significantly different from the control.

To evaluate the mechanism of PMCTi‐induced apoptosis, ROS generation, including hydrogen peroxide, hydroxyl radicals, and peroxynitrite, was quantitated by flow cytometry. As shown in Fig. 5A, after treatment with 2.5 mm PMCTi for 24 h, the level of ROS generation in HepG2 cells was increased to 1316.7% relative to 100% of the control. In contrast, primary human hepatocytes were not significantly affected in ROS generation after PMCTi treatment. Figure 5B shows increased ROS levels in HepG2 cells stained with CM‐H_2_DCFDA and detected by confocal microscopy. ROS levels are shown in the green channel; cells treated with 2.5 mm PMCTi increased in fluorescence intensity when compared with 0.2% DMSO as a control. This result indicates that PMCTi induces ROS production that might lead to a contribution to apoptosis in HepG2 cells.

To determine whether a depletion of the DNL pathway also played a role in triggering the extrinsic apoptotic pathway, which also causes disruption of ∆Ψm, this study measured caspase‐8 activity after treatment with 2.5 mm PMCTi for 24 h. The effect of PMCTi on caspase‐8 activity of HepG2 cells was analyzed by the FLICE/Caspase‐8 Fluorometric Assay Kit. Our results showed that caspase‐8 activity significantly increased to 130% relative to 100% of control cells following PMCTi treatment. These findings indicate that PMCTi depletes the DNL pathway causing apoptosis in HepG2 cells through the extrinsic and intrinsic mitochondrial‐dependent pathway.

## Discussion

In cancer cells, the upregulated DNL pathway is characterized as a metabolic hallmark that supports enhanced cell growth and proliferation. This pathway begins the conversion of cytosolic citrate to acetyl‐CoA which then provides LCFAs products for a variety of cellular processes 43. Fatty acids essentially benefit by supplying precursors for biosynthesis of cell membranes, anchors of membrane target proteins, signaling second messengers, and ATP production via β‐oxidation 44. In the liver, the level of citrate is well maintained via both mitochondrial membrane CTP exported from the mitochondria TCA cycle and plasma membrane PMCT taken up from circulation 22, 28. PMCT expression has been discovered in *D. melanogaster* and *C. elegans*. Reduction in the analogue gene expression in both fruit flies and worms surprisingly causes an increase of average life span as a result of decreasing body size and fat content 20, 26. Beyond this former discovery, results obtained from mice were in accordance in that suppression of mammalian PMCT contributes to reduction in hepatic lipid accumulation, amelioration of hepatic insulin sensitivity, and prevention of diet‐induced nonalcoholic fatty liver disease in high‐fat diet‐induced mice 27. Therefore, PMCT plays a role in incorporating citrate for fatty acid synthesis, accumulation of which is rather disadvantageous in normal cells. However, recent study has revealed that depletion of SLC13A5 or PMCT results in reduction of cell proliferation in HepG2 and Huh7 cancer cells. This reduction is correlated with a decrease in the DNL pathway 45. Thus, inhibition of citrate transport by PMCTi targeting the suppression of DNL synthesis is hypothesized to be a novel therapeutic approach in cancer cells.

In the present study, the results showed that PMCTi decreased cell viability and induced apoptosis in HepG2 cells. We demonstrated that apoptosis induction by PMCTi is associated with inhibition of the DNL pathway, illustrated by decreasing intracellular citrate, fatty acid, and triacylglycerol levels. We obtained a consistent result from C75, a pharmacologic inhibitor of FASN in the DNL pathway; that inhibition of the DNL pathway promoted apoptosis in cancer cells 46. However, inhibition of PMCTi showed no alteration of abundance of expression of FASN, ACC, and ACLY proteins responsible for the DNL pathway. As a result, inhibition of citrate transport consequently caused suppression of fatty acid synthesis in cancer cells. Apoptosis is influenced by the depletion of fatty acid precursors for the synthesis of membrane phospholipid during cell proliferation 47. Phosphatidylcholine, the major membrane phospholipid in eukaryote cells, which is synthesized from the endogenous fatty acid synthesis, accounts as the precursor for other major phospholipid biosynthesis in proliferating cells, including sphingomyelin and phosphatidylethanolamine 48. Moreover, a recent study has demonstrated that reduction in phosphatidylcholine results in elevation of ceramide levels, which is a potent inducer of apoptosis 49.

Depletion of fatty acid levels is known to cause an accumulation of malonyl‐CoA levels. This accumulation generates an inhibitory effect on CPT‐1 activity which induces apoptosis in cancer cells 39, 42, 50. In fact, newly synthesized fatty acid evokes negative feedback on malonyl‐CoA levels by inhibiting ACC enzyme activity 51. Depletion of fatty acid by PMCTi thus provides a weaker inhibition on ACC activity which then elicits an accumulation of malonyl‐CoA level. These high malonyl‐CoA levels will trigger an inhibition of CPT‐1 activity 39. We found that an inhibition of ACC activity by TOFA alleviated apoptosis mediated by dissipation of ΔΨm by PMCTi and brought mitochondrial membrane potential closer to normal. In the meantime, decreased CPT‐1 activity by exposure of PMCTi in HepG2 cells represents an inhibitory result of an accumulation of malonyl‐CoA level. We suggest that a decrease in CPT‐1 activity as a result of suppressed DNL by PMCTi can be considered an inducer of apoptosis in HepG2 cells. We also found that the apoptotic effect of C75 possibly mediates through other pathways rather than inhibition of CPT‐1 activity. Instead, C75 directly stimulates CPT‐1 activity 41, 42.

There is a positive correlation between CPT‐1 activity and acetyl‐CoA end products from fatty acid β‐oxidation which continues to replenish citrate for the TCA cycle. CPT‐1 transesterifies fatty acyl CoA to acylcarnitines. After being transported into the mitochondria matrix by carnitine acylcarnitine translocase, acylcarnitines are transesterified back to fatty acyl CoA by CPT‐2 for fatty acid β‐oxidation 38. Inhibition of CPT‐1 enhances synthesis of ceramide from an accumulation of fatty acyl CoA instead of converting into acetyl‐CoA 52, 53, 54. Ceramide has an apoptosis induction role in cancer cells through activation of various pro‐apoptotic molecules, such as Bcl‐2 nineteen‐kilodalton interacting protein 3 (BNIP3), a tumor necrosis factor‐related apoptosis‐inducing ligand (TRAIL) 55, and death‐associated protein kinase 2 (DAPK2) 56.

Notably, our result showed that inhibition of CPT‐1 activity is correlated with enhanced loss of ΔΨm and increased caspase‐8 activity following PMCTi treatment in HepG2 cells. Previous studies report that the reduction in fatty acids such as palmitate and their cognate CoA derivatives promotes the opening of the mitochondrial permeability transition pore to bind to the adenine nucleotide translocator and to release cytochrome c for the apoptosis process 57, 58, 59. Ceramide also enhances cells sensitized to TRAIL‐induced apoptosis in renal cell carcinoma cells 60. Binding of TRAIL to TRAIL receptors facilitates recruitment of the adaptor protein FADD and procaspase‐8 which is then cleaved to active caspase‐8 61, 62. Cleaved caspase‐8 consequently activates the apoptotic protein Bid, which leads to the triggering of mitochondrial damage, which in turn induces apoptosis 61. Taken together, decreased fatty acid synthesis resulting from PMCTi treatment inhibits CPT‐1 activity which consequently promotes apoptosis via activation of both mitochondrial damage and caspase‐8 activity. Surprisingly, under PMCTi treatment, mitochondrial damage and apoptosis were not observed in primary human hepatocytes, which was comparable to a previous study which reported that depletion of the DNL pathway by inhibiting enzymes in the DNL pathway has no apoptosis effect in normal cells 63, 64. Less expression of DNL proteins in normal cells represents less activity in fatty acid synthesis where inhibitors have less effect when compared with cancer cells.

In addition, it has been reported that knockdown of PMCT results in a significant decrease in intracellular levels of the ATP/ADP ratio, which consequently activates the cellular energy sensor AMP‐activated protein kinase (AMPK). This protein activation signals deactivation of the oncogenic mechanistic target of rapamycin (mTOR) signaling, leading to attenuating the growth of HCC cells 45. The level of ATP production via fatty acid β‐oxidation is considered a great power in stimulating tumor growth 65. Pharmacological inhibitors of β‐oxidation generate a reduction in the ATP supply that causes impairment of cell proliferation reported in PC3 prostate cancer 66 and human leukemia cells 16. Thus, we suggest that reduced fatty acid β‐oxidation as a result of inhibition of CPT‐1 activity by PMCTi could lead to regulation of the ATP/ADP‐activated AMPK/mTOR pathway. Finally, the cellular proliferation pathway is simultaneously suppressed.

Several studies have reported that decreased fatty acid production also enhances ROS generation, resulting in reduction in the proliferation and activation of apoptosis in cancer cells 31, 67. Our results found increased ROS generation correlated to decreasing fatty acid production in HepG2 cells. Taken together, we suggest another mechanism by which PMCTi induces apoptosis through enhanced ROS generation. Suppression of CPT‐1 activity facilitates increased ROS generation through impairment of NADPH, a potent nonenzymatic antioxidant in mitochondria, in human glioblastoma cells, and subsequently drives ATP depletion, finally inducing apoptosis cell death 40.

In conclusion, our findings demonstrate that the inhibition of the DNL pathway by suppression of citrate transport by PMCTi induces apoptosis through the mitochondrial‐dependent pathway in HepG2 cells. This result will potentially assist in the development of novel cancer treatment that targets the inhibition of the DNL pathway. In addition, targeting DNL by suppression of citrate transport pathways will be one of the therapeutic interventions of metabolic disorders resulting from the synthesis of excess lipids in human obesity, hyperlipidemia, hyper‐cholesterolemia, and type 2 diabetes. However, all of the data obtained from this study addressing the effect of PMCT inhibition on the citrate transport pathway were based on studies performed only in the HepG2 cell line. These findings may not translate to other types of cancers and currently limit the application of this inhibitor being a new targeted anticancer therapy. Therefore, more types of cancer cell line models will be required to sufficiently interpret for the most widely preclinical targeted drug model.

## Author contributions

WP, PP, and PS conceived and designed the experiments. PS supervised the project. WP, PP, NP, and SS performed the experiments. WP and PS analyzed the data. WP, PP, WK, DP, LR, and PS contributed reagents/materials/analysis tools. WP, PP, DP, and PS wrote the manuscript. All authors read and approved the final manuscript.
